# Automated Anatomical Landmark Localization in Anterior Segment OCT Images Using an Efficient Deep Learning Framework

**DOI:** 10.3390/s26154982

**Published:** 2026-08-06

**Authors:** Liangqi Zheng, Yingping Deng, Zhiyong Huang, Jing Tang, Li Chen

**Affiliations:** 1 School of Aeronautics and Astronautics, Sichuan University, Chengdu 610207, China; lq_zhengscu@163.com; 2 Ophthalmology Department of West China Hospital, Sichuan University, Chengdu 610041, China; lan15806599686@163.com (Y.D.); 13795650299@163.com (L.C.)

**Keywords:** optical coherence tomography (OCT), pose-point estimation, YOLOv11 architecture, attention mechanism, lightweight network

## Abstract

Anterior segment optical coherence tomography (AS-OCT) is essential for structural assessment of the anterior eye, yet automated landmark localization remains challenged by pervasive speckle noise, indistinct tissue interfaces, and labor-intensive manual annotation with notable inter-observer variability. This study presents NSE YOLO, an enhanced YOLOv11 framework for high-precision landmark localization in AS-OCT images after implantable collamer lens (ICL) implantation. It integrates a dual-branch NewConv module for multi-scale feature extraction, a dual-additive residual self-attention block (SABlock) to suppress background interference, and a Mamba-based EfficientViMBlock embedded in the C3k2 module to balance global contextual modeling and computational efficiency. Validated on 672 expert-annotated postoperative ICL images from 60 patients, NSE YOLO achieved an mAP@0.5 of 90.7% and mAP@0.5:0.95 of 85.1%, outperforming the YOLOv11 baseline by 5.2% and 6.6% with only 2.86 million parameters. Bland–Altman analysis showed negligible systematic bias and narrow limits of agreement for anterior chamber depth. For iridocorneal angle measurements, directional deviations and wider limits of agreement were observed, with performance approaching the level of inter-observer variability among human annotators. NSE YOLO enables automated quantification of anterior chamber depth and bilateral iridocorneal angles for post-ICL follow-up assessment, providing preliminary technical validation supporting further external and device-level evaluation.

## 1. Introduction

Optical coherence tomography (OCT) has revolutionized ophthalmic diagnostics by providing real-time, non-invasive, high-resolution cross-sectional imaging of ocular structures [1,2,3,4]. For example, advanced modalities like OCT angiography (OCTA) can objectively evaluate retinal microcirculation and serve as an effective technology to track the treatment response of patients with ocular conditions such as Vogt–Koyanagi–Harada disease [5]. It provides real-time, in vivo three-dimensional structural imaging with micrometer-level resolution, effectively bridging the technical gap between macroscopic imaging and invasive histopathological biopsies [6,7,8,9]. Anterior segment optical coherence tomography provides non-contact cross-sectional visualization of the cornea, anterior chamber, iris, crystalline lens, and iridocorneal angle [10,11]. Quantitative analysis of these structures supports anterior-segment morphometry and angle assessment, including the identification of narrow or closed angles [12,13,14]. In corneal and refractive surgery, OCT-derived structural measurements can inform preoperative assessment, treatment planning, and postoperative evaluation [11,15,16,17]. Intraoperative OCT provides cross-sectional information on tissue depth and interfaces during corneal surgery, particularly lamellar keratoplasty, and can support intraoperative assessment and surgical decision-making [18,19,20]. Reliable landmark localization underpins these quantitative applications. In the present study, the scleral spur and angle apex provide the geometric references for bilateral iridocorneal-angle measurement, whereas the anterior and posterior corneal surfaces and the anterior lens surface define the anterior chamber axis used to derive anterior chamber depth.

Despite its profound clinical utility, the automated interpretation of AS-OCT images is severely hindered by both physical and technical bottlenecks. The inherent inverse correlation between imaging resolution and tissue penetration depth inevitably results in signal roll-off and blurred structural boundaries at deeper tissue interfaces [21,22,23,24], such as the scleral spur and ciliary body. Furthermore, AS-OCT imaging is highly susceptible to intrinsic speckle noise, which creates irregular mottled patterns, and motion artifacts induced by physiological micro-saccades [22,23,24,25,26,27,28]. Consequently, landmark annotation remains heavily reliant on the subjective experience of clinicians. This manual process is not only labor-intensive and unsuitable for high-throughput clinical workflows but also introduces significant inter-observer variability, underscoring the urgent need for robust, automated localization algorithms [29,30,31,32,33,34,35].

Recent advancements in deep learning have catalyzed the transition toward automated ophthalmic image analysis, with object detection architectures primarily bifurcating into two-stage and single-stage models [36,37,38,39]. Two-stage detectors, such as the R-CNN [40,41,42] family and Faster R-CNN [43,44,45,46,47], achieve high localization accuracy through region proposal mechanisms but suffer from substantial computational overhead, rendering them suboptimal for high-throughput clinical workflows. Conversely, single-stage architectures have emerged as the preferred paradigm by directly regressing bounding boxes and class probabilities. Among these, the You Only Look Once (YOLO) [48] series has established itself as a robust and highly reliable baseline for medical image analysis. Compared to other single-stage models like SSD [49] or RetinaNet [50], YOLO leverages a unified, globally contextualized network design that inherently balances rapid inference speeds with high detection accuracy. The latest iteration, YOLOv11, further solidifies this reliability by incorporating advanced spatial pyramid pooling (SPPF) and enhanced feature extraction modules (such as C3k2 and C2PSA), granting it exceptional versatility in multi-scale object detection [51,52,53]. YOLO architectures have emerged as the preferred backbone for real-time object detection tasks, achieving a practical balance between accuracy and efficiency. In the context of medical imaging, compared to traditional manual visual inspections that are labor-intensive, subjective, and prone to overlooking subtle features, the YOLOv11 algorithm offers automated localization of clinically relevant regions. However, standard YOLO models struggle to fully capture small objects against low-contrast backgrounds, often resulting in biased detection outcomes. Furthermore, optical coherence tomography (OCT) images inherently suffer from speckle noise, low contrast, and complex tissue backgrounds, which significantly exacerbate feature uncertainty during the extraction process [54,55].

To bridge this translational gap, this study proposes NSE YOLO, a highly refined deep learning framework tailored specifically for robust landmark and object detection in AS-OCT images. By progressively optimizing the YOLOv11 architecture, we aim to deliver precise, automated localization of multi-scale, blurred-boundary anatomical structures without compromising inference speed. The main contributions of this study are systematically aligned with resolving critical clinical imaging challenges:

**(1)**  **NewConv Module for Mitigating Feature Uncertainty:** To address the severe speckle noise and fuzzy boundaries in AS-OCT, we introduce the NewConv module into the backbone network. By integrating multi-scale feature aggregation with a multi-head parallel architecture, this module effectively suppresses intrinsic artifacts while preserving the precise spatial representation of minute anatomical landmarks, significantly enhancing the model’s diagnostic reliability.
**(2)**  **SABlock Module for Complex Background Suppression:** The anterior chamber angle and deeper structural regions often present as dark, low-contrast backgrounds that degrade pose detection accuracy. We design a Self-Attention Block (SABlock) utilizing a dual-additive residual architecture. This mechanism captures long-range contextual dependencies, effectively filtering out spatial noise and amplifying the focus on critical landmark features amidst complex tissue backgrounds.
**(3)**  **C3k2_EVM Module for Computational Efficiency:** To reduce model complexity while retaining global contextual modeling, we embed the EfficientViMBlock within the C3k2 module of the neck network. Leveraging the lightweight Mamba architecture and state-space duality, this design optimizes primary feature processing, striking an optimal balance between high-fidelity spatial computation and minimal algorithmic latency.

Through extensive validation on an expert-annotated clinical AS-OCT dataset reflecting diverse real-world challenging scenarios (e.g., low contrast, ambiguous boundaries, missing regions). This study evaluates the localization accuracy, computational efficiency, and internal robustness of NSE YOLO on an expert-annotated AS-OCT dataset.

## 2. Methods

### 2.1. Preparation of the Imaging Datasets

**Figure 1:**
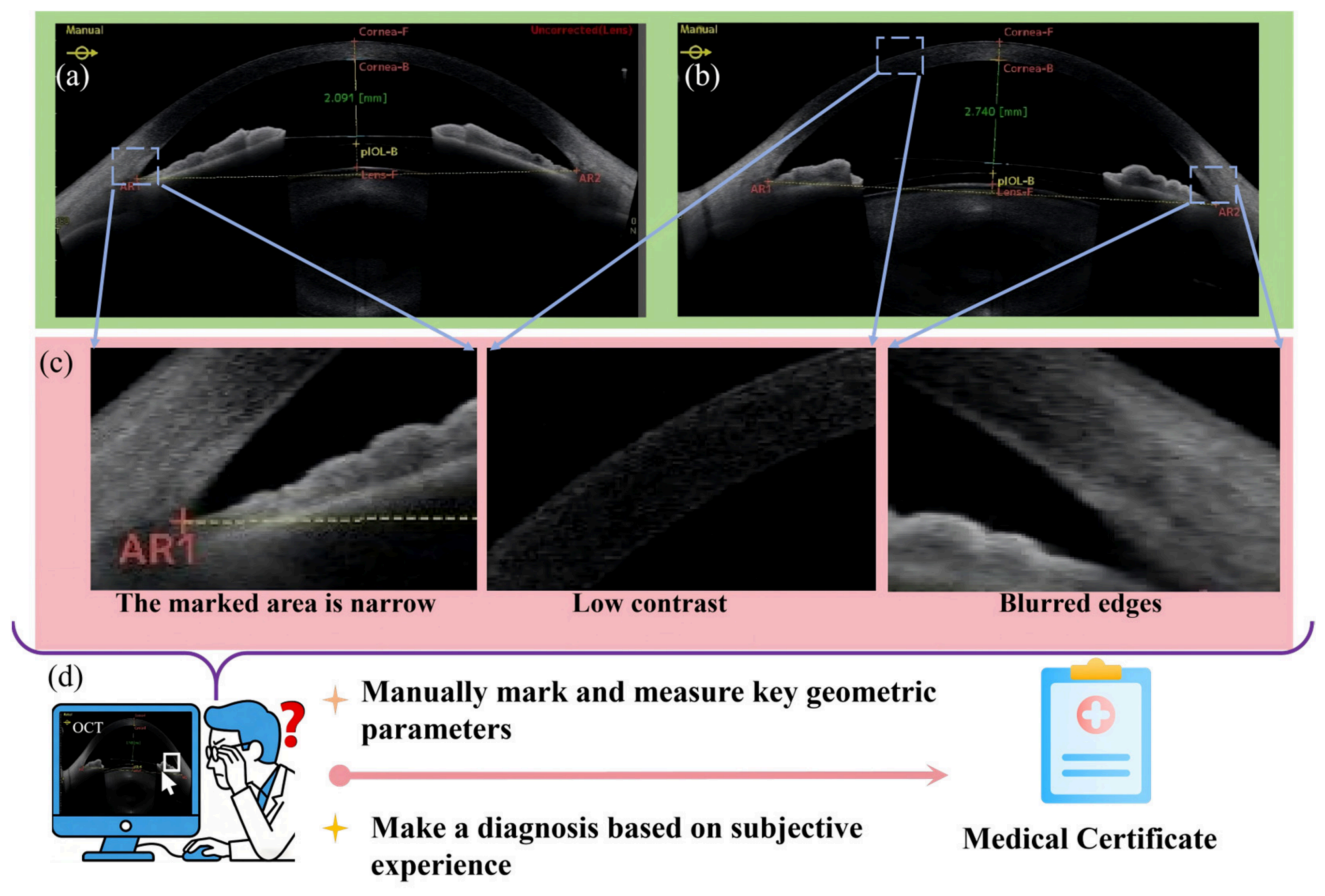
Examples of OCT image datasets and the challenges they present: (**a**) anterior chamber OCT image of narrow IAs; (**b**) anterior chamber OCT image of normal IAs; (**c**) the common challenges encountered in OCT imaging include narrow regions of interest, low contrast, and unclear edges; (**d**) following the manual marking and measurement of key geometric parameters by an ophthalmologist, such as the IAs and anterior chamber depth, a diagnosis is made based on subjective experience. The green background marks the raw OCT image display area, and the pink background marks the imaging challenge demonstration area. Blue bounding boxes and connecting callout lines indicate the correspondence between the regions of interest in panels (**a**)/(**b**) and their magnified sub-images in panel (**c**). The pink horizontal arrow in panel (**d**) denotes the progression direction of the clinical diagnosis workflow.

All collected OCT images undergo uniform edge cropping to ensure consistent resolution at 924 × 456 pixels, as shown in Figure 1. During cropping, it is essential to preserve image integrity while ensuring that patient privacy information, markers and other irrelevant areas are removed. Following cropping of the images, annotate the posture points of the iridocorneal angle (IAs) and the anterior chamber axis on OCT images within the LabelME platform, ensuring high-quality annotations and accuracy for the training samples. This study enrolled a uniform postoperative cohort without preoperative phakic eyes or eyes with other types of intraocular lenses. All performance evaluations were conducted exclusively on AS-OCT images from eyes after ICL surgery to ensure clinical relevance to postoperative follow-up scenarios. In view of the limited proportion of clinically challenging cases among AS-OCT images obtained after ICL surgery, data augmentation techniques, including horizontal flipping, synchronous rotation of images and keypoints, brightness and contrast adjustment, and Gaussian noise addition, were applied in RoboFlow v1.0 (https://roboflow.com) to expand the training dataset. This approach reduces the cost of raw data acquisition and the risk of overfitting while enhancing the learning capability and generalization performance of deep learning models. The total number of images acquired was 672, with 80% allocated for training, 10% for validation, and the remainder for testing.

The dataset was acquired at a single center using a CASIA2 anterior segment swept-source OCT system (Tomey Corporation, Nagoya, Japan) and comprised 672 images from 60 patients. All images were exported at a native resolution of 924 × 456 pixels, corresponding to an approximate pixel spacing of 0.0133 mm/px. Eligible participants were adults undergoing routine postoperative follow-up after ICL implantation. An image was included when the cornea, anterior chamber, and at least one iridocorneal angle were fully captured within the field of view. Scans with severe motion artifacts, complete signal attenuation, or missing regions of interest were excluded. Cases with clinically challenging features, including narrow iridocorneal angles, low-contrast tissue interfaces, oblique scanning sections, and prominent vault effects, were deliberately retained to preserve clinical heterogeneity within the postoperative cohort. Ground-truth landmarks were established under a predefined annotation protocol.

Ground-truth landmarks were established under a predefined annotation protocol. Each anatomical landmark was explicitly defined prior to labeling. Three keypoints were annotated for each iridocorneal angle, namely the posterior corneal reference point, the scleral spur as the angle vertex, and the anterior iris reference point. Two axial landmarks were labeled for anterior chamber depth calculation, corresponding to the midpoint of the posterior corneal surface and the midpoint of the anterior lens surface. All images were independently annotated by three ophthalmologists with extensive clinical experience. The coordinate-wise mean of the three annotations was adopted as the final reference standard. Cases with pronounced inter-annotator divergence were resolved through consensus discussion.

A native pixel spacing of 0.0133 millimeters per pixel was used to convert pixel distances to physical units. Anterior chamber depth was calculated as the axial Euclidean distance between the two central landmarks along the anatomical axis of the anterior chamber. For obliquely oriented scans, landmarks are localized strictly along the central anatomical axis rather than the vertical axis of the image plane. This axial definition of central anterior chamber depth conforms to the standardized measurement protocol in clinical AS-OCT biometry [56,57], which ensures measurement validity independent of scan tilt. Each iridocorneal angle was derived from its three corresponding keypoints with standard three-point angle geometry. The scleral spur serves as the fixed anatomical vertex of the angle. The corneal reference point is located on the posterior corneal surface immediately anterior to the scleral spur, and the iris reference point is located on the anterior iris surface immediately posterior to the scleral spur. The iridocorneal angle is defined as the included angle formed by the two line segments connecting each reference point to the scleral spur. This three-point landmark protocol is consistent with the standardized AS-OCT angle measurement convention widely adopted in clinical practice [14,58,59].

Inter-observer variability was quantified on the test set as the mean pairwise Euclidean distance between independent annotations. The metric was normalized with the same reference length applied to model error calculation to enable direct comparison. Full quantitative assessment of inter- and intra-observer reliability across the full dataset lies outside the scope of this work. A targeted comparison on the test set is provided in Section 3.5, and this point is revisited as a limitation in the Discussion.

To prevent information leakage, the dataset was partitioned at the patient level into training (80%), validation (10%), and test (10%) subsets. All images belonging to a given patient—including both eyes and repeated acquisitions—were assigned to a single subset, so that no patient contributed images to more than one partition. This patient-independent partitioning ensures that the reported performance is not inflated by identity leakage across subsets. In addition to the single hold-out test set, patient-level five-fold cross-validation was performed to assess robustness to data partitioning. Data augmentation was applied independently within the training subset of each fold. No patient contributed images to more than one fold across all iterations to eliminate identity leakage.

Data augmentation was applied strictly after dataset partitioning and exclusively to the training subset. The augmentation pipeline included horizontal flipping, rotation within a range of ±15 degrees, brightness and contrast adjustment, and Gaussian noise addition. All geometric transformations were applied synchronously to both images and keypoint coordinates. Each original image and all its augmented variants remained within the same subset. The validation and test subsets contained only original, non-augmented images.

**Figure 2:**
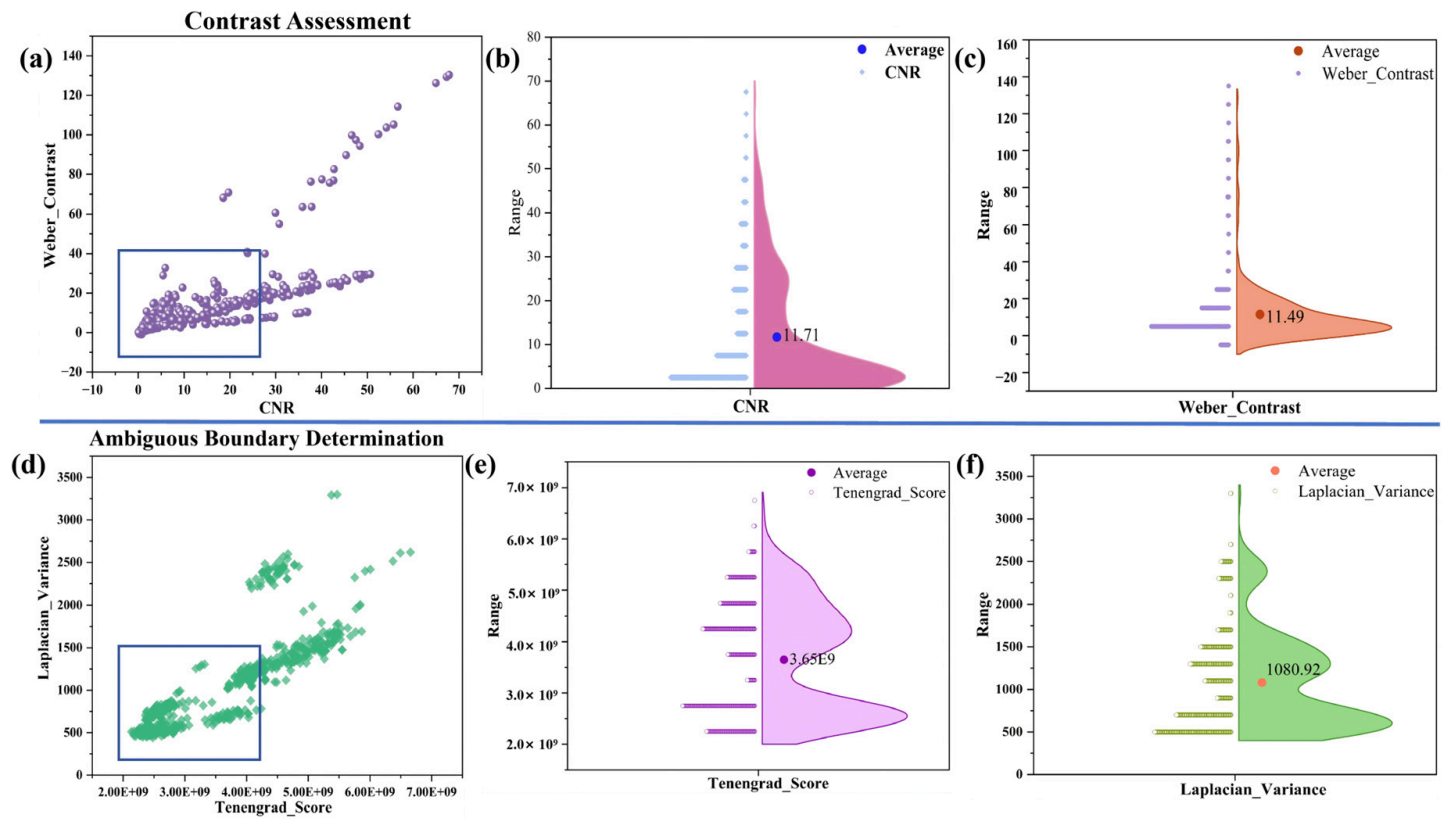
Quantitative evaluation distribution of contrast and boundary ambiguity in the anterior segment training set. (**a**) Joint scatter plot of the contrast-to-noise ratio (CNR) and Weber contrast, with the solid blue box highlighting the core clustering region of low-contrast samples; (**b**) raincloud plot illustrating the distribution and mean value (11.71) of CNR; (**c**) raincloud plot illustrating the distribution and mean value (11.49) of Weber contrast; (**d**) joint scatter plot of the Tenengrad score and Laplacian variance, with the solid blue box indicating the clustering region of samples with severe boundary ambiguity; (**e**) raincloud plot illustrating the distribution and mean value (3.65 × 10^9^) of the Tenengrad score; (**f**) raincloud plot illustrating the distribution and mean value (1080.92) of the Laplacian variance.

The dataset encompasses a range of scenarios, including low contrast, blurred edges, and background noise. To quantify the image characteristics of the training set, this study evaluated its contrast and boundary blurriness, as shown in Figure 2. The analysis indicates that the contrast-to-noise ratio (CNR) and Weber contrast, with means of 11.71 and 11.49, respectively, as well as the Tenengrad score and Laplacian variance (mean 1080.92), all exhibit right-skewed distributions. The data samples primarily cluster in regions of low contrast and low gradient values. This distribution reflects the speckle noise and local defocusing commonly observed in anterior segment imaging. Retaining these unfiltered, naturally distributed samples aims to reduce the risk of deep learning models overfitting to high-SNR data, providing testing conditions that more closely resemble actual clinical scenarios for evaluating the generalization ability of the proposed YOLOv11 framework.

**Figure 3:**
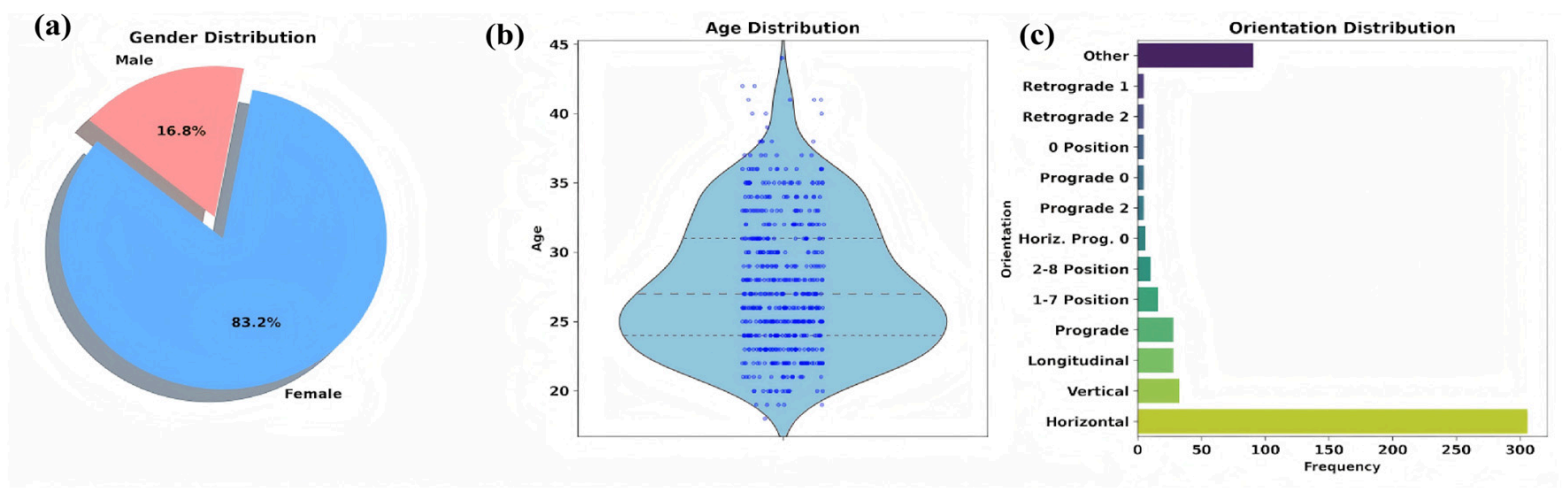
Distribution characteristics of demographics and acquisition conditions in the anterior segment training set. (**a**) Pie chart of gender distribution within the samples. (**b**) Raincloud plot depicting the age distribution, where the light blue filled area represents the kernel density contour of the age distribution, and the three grey dashed lines indicate the 25th, 50th (median), and 75th percentiles of age, respectively. (**c**) Bar chart of the frequency of imaging orientations.

This study quantitatively analyzed the baseline characteristics of demographics and imaging conditions in the training set (Figure 3). As shown in Figure 3a, the dataset exhibits a significant gender distribution discrepancy, with a substantially higher proportion of female samples compared to male samples. The age distribution presents right skewness (Figure 3b), with samples primarily concentrated in young and middle-aged groups, and this data characteristic provides a testing condition for evaluating the model’s generalization ability to degenerative physiological structures in elderly patients. Furthermore, the imaging orientations present a long-tailed distribution (Figure 3c), where samples mostly cluster in the standard horizontal section with limited non-standard posture data. This spatial geometric imbalance increases the network’s difficulty in processing non-rigid tissue deformations and performing feature alignment. Retaining this raw skewed distribution as the training baseline aims to reflect the natural acquisition state of clinical data, establishing an objective foundation to evaluate the feature extraction robustness of the proposed object detection algorithm under imbalanced conditions.

### 2.2. Experimental Environment and Evaluation Parameters

The experiments in this study were conducted on a Windows 11 operating system, with an Intel(R) Xeon(R) Gold 6330 CPU and an RTX 3090(24 GB) GPU. The software environment included Python 3.8 (ubuntu20.04), CUDA 11.8, and PyTorch 2.0.0. The experimental parameters are shown in Table 1.

This study conducts a comprehensive performance evaluation of the YOLO series of models, with the core evaluation metrics being mAP50%, mAP50-95%, and Params. The first metric measures basic detection accuracy, the second metric measures comprehensive localization performance, and the third metric measures model complexity. Specifically, all reported mAP values describe bounding-box detection performance for target anatomical regions, including the anterior chamber region and bilateral iridocorneal angle regions. mAP50% is defined as the mean average precision across all region categories at an intersection over union threshold of 0.5. The metric reflects basic region localization capability and robustness against imaging interference, serving as the core benchmark for object-level detection performance. The average of multi-threshold average precision across IoU thresholds ranging from 0.5 to 0.95 in 0.05 increments is denoted mAP50-95%. This metric primarily measures fine-grained bounding-box regression accuracy and overall region detection quality. Keypoint localization accuracy is evaluated independently via pixel-wise errors, normalized mean error, and clinically interpretable metrics. Params refers to the number of model parameters, used to quantify the total scale of learnable parameters, and serves as a key basis for evaluating inference efficiency and deployment adaptability. The three types of metrics under discussion complement one another, thereby enabling an objective evaluation of model performance and lightweight design. Furthermore, they provide quantitative support for model optimization, selection, and practical applications.

Bounding-box matching for mAP calculation relies entirely on intersection over union between predicted and ground-truth bounding boxes. Keypoint localization performance is assessed separately using Euclidean distance between predicted and reference coordinates, normalized to normalized mean error with a unified reference length. The two evaluation systems operate independently, and keypoint accuracy is not incorporated into mAP computation.

True Positive (*TP*) refers to the number of correctly predicted positive samples, False Positive (*FP*) refers to incorrectly predicted positive samples, and False Negative (*FN*) indicates the number of missed positive samples. Precision (*P*) and Recall (*R*) are calculated as Equations (1) and (2):

(1) $$P\;=\;\frac{\mathrm{TP}}{\mathrm{TP}\;+\;\mathrm{FP}}$$

(2) $$R=\frac{\mathrm{TP}}{\mathrm{TP}+\mathrm{FN}}$$

Average Precision (*AP*) is the area under the precision–recall curve, shown in Equation (3):

(3) $$AP=\int_{0}^{1}P(R)dR$$

*mAP50%* and *mAP50–95%*, are computed as defined in Equations (4) and (5), respectively.

(4) $$mAP50\%=\frac{1}{N}\sum_{i=1}^{N}{AP}_{0.5,i}$$

(5) $$\;mAP50–95\%=\frac{1}{10N}\sum_{t=0.5}^{0.95}\sum_{i=1}^{N}{AP}_{t,i}$$

Besides computer-vision detection metrics, clinically interpretable metrics were adopted to evaluate the practical diagnostic value of the model. Mean absolute error (MAE), median absolute error and the standard deviation (Std) of absolute errors were calculated to quantify the magnitude of measurement deviation between automated predictions and the expert reference standard. Clinically acceptable error thresholds were defined as 0.2 mm for ACD and 5° [56,57,58,59] for IA, based on the reported repeatability of manual AS-OCT measurements and the step width of the standard Shaffer angle grading system, to ensure no misclassification of clinical risk stratification. Bland–Altman analysis was performed to assess systematic bias and 95% limits of agreement (LoA); the 95% confidence interval (CI) of the mean bias was calculated using the t-distribution, and 95% LoA were defined as the mean bias ± 1.96 × the standard deviation of paired differences. Samples were further stratified by ground-truth ACD and IA width to evaluate model robustness across different clinical difficulty levels.

### 2.3. Optimization Strategies for YOLOv11n

**Figure 4:**
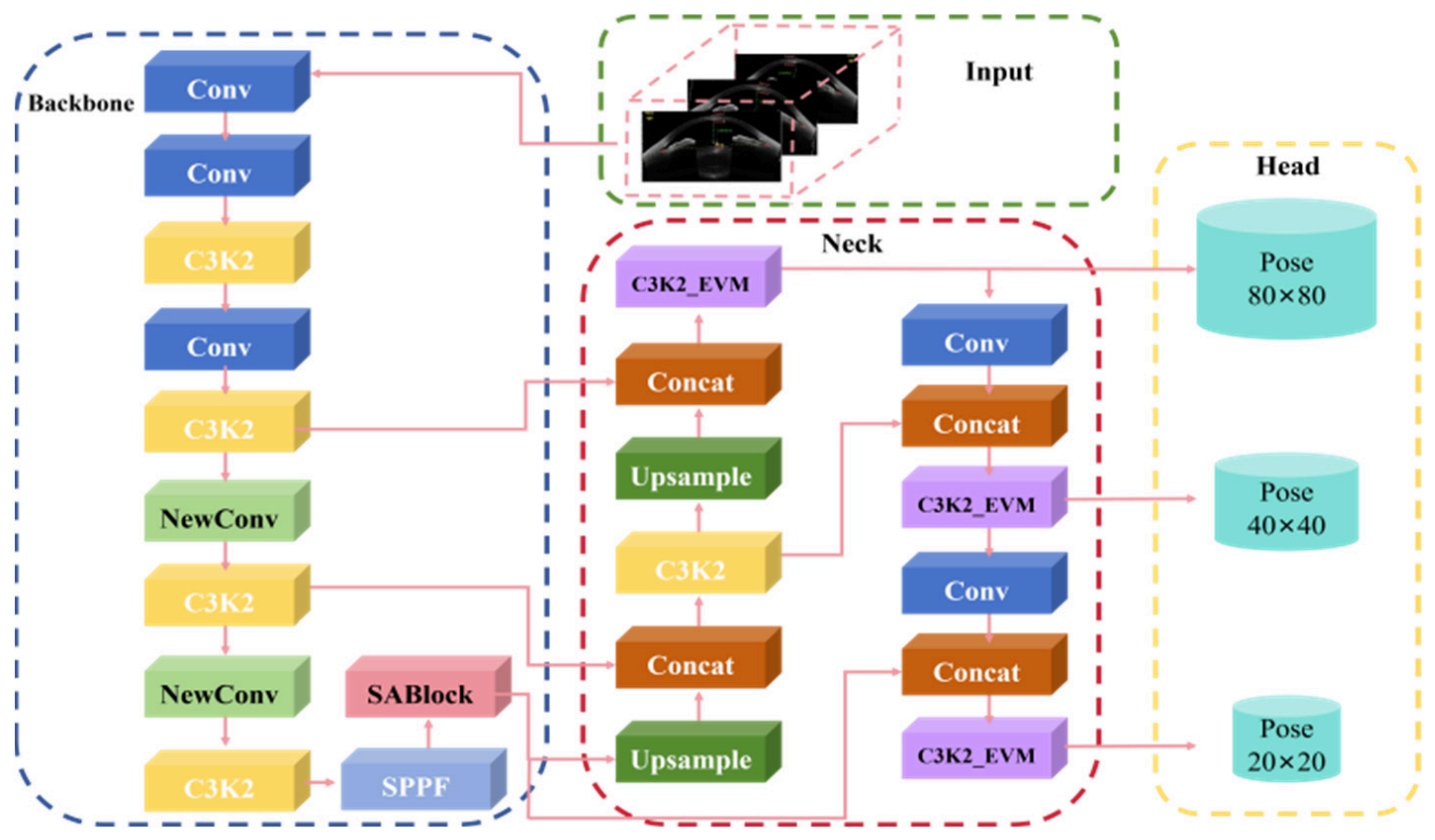
Improved YOLOv11 network structure. Dashed boxes in different colors divide the whole network into four functional modules: the green dashed box represents the input module, the blue dashed box represents the backbone feature extraction network, the red dashed box represents the neck feature fusion network, and the yellow dashed box represents the pose detection head. Red arrows indicate the flow direction of feature data, including forward propagation paths and cross-scale skip connections.

The OCT image pose point detection model based on an improved version of YOLOv11 is illustrated in Figure 4.

#### 2.3.1. NewConv Model

**Figure 5:**
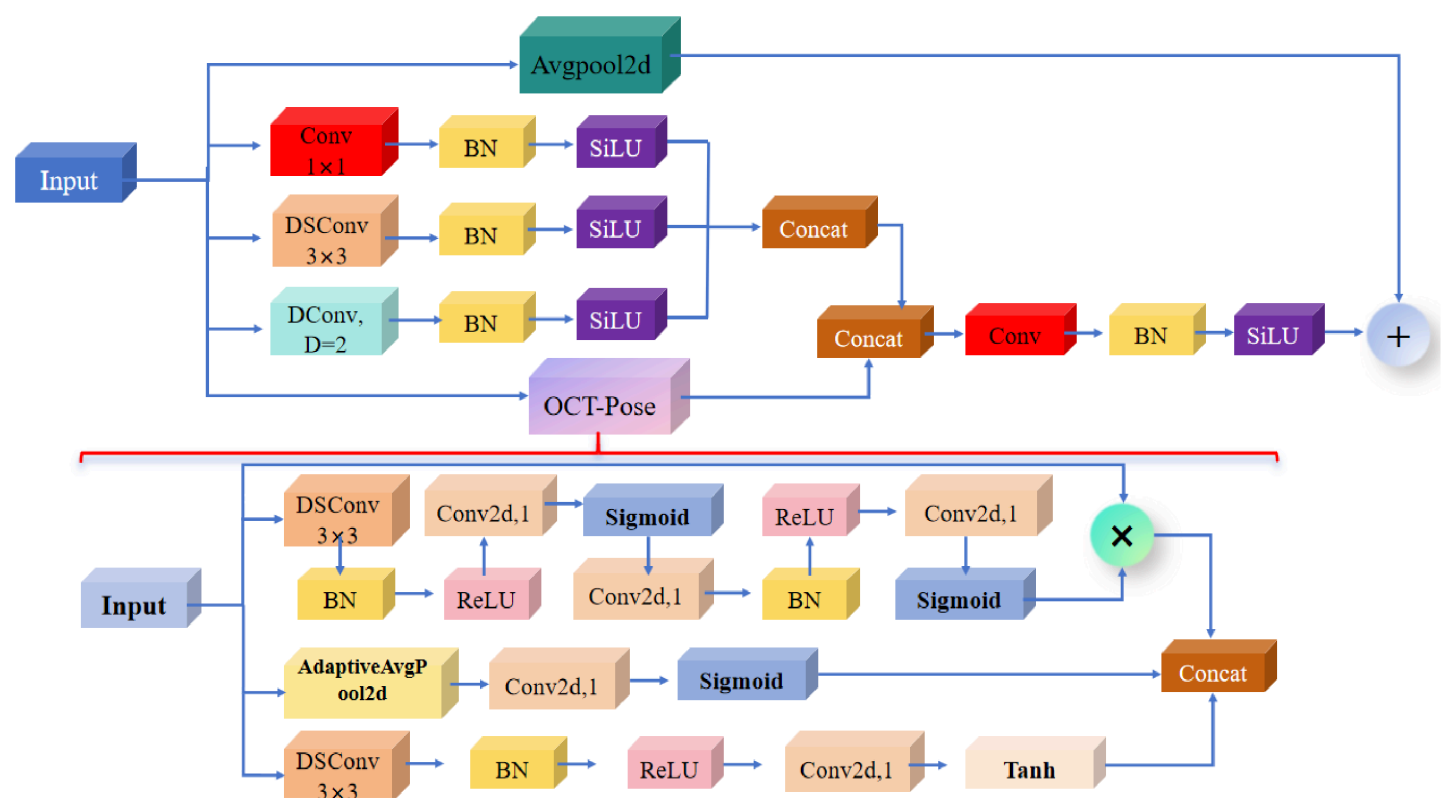
NewConve model network structure diagram. Blue arrows indicate the forward propagation direction of feature data, covering multi-branch feature extraction paths and residual shortcut connections. The red horizontal line demarcates the internal detailed architecture of the OCT-Pose module in the lower region.

In order to calculate edge angle and midline distance in OCT image detection, enhanced pose point detection is required. The NewConv pose point detection module was designed with two branches, as illustrated in Figure 5. The input feature map is defined as ***X*∈R^H×W×C^**, where **H**, **W**, and **C** represent the height, width, and channel number of the feature map, respectively. The first branch employs a multi-scale feature extraction and fusion approach: 1 × 1 convolutions (**Conv**) are used to capture local texture details; 3 × 3 depth-separable convolutions (**DSConv_3×3_**) are used to extract mesoscale shape variations; and deformable convolutions (**DConv**) are employed to obtain a larger receptive field for identifying global deformation features, followed by channel concatenation to complete multi-scale feature fusion. This approach resolves the feature adaptation issue for keypoints at different scales—such as the IA and the cornea—in OCT images, ensuring robust processing of pose point features. This process is represented by Equation (6).

(6) $$F_{\mathrm{multi}}=\mathrm{Concat}\;(\sigma (\mathrm{BN}(\mathrm{Conv}(X))),\;\sigma (\mathrm{BN}(\mathrm{DSConv}(X))),\;\sigma (\mathrm{BN}(\mathrm{DConv}(X))))$$

where **BN**(·) is batch normalization, ***σ***(·) is the SiLU activation function, and **Concat**(·) is channel-wise concatenation.

The second branch is the pose detection module, which employs a multi-head parallel architecture to precisely locate key anatomical landmarks of the eye. This branch incorporates a co-attention mechanism to suppress dark-background noise and enhance feature responses of faint targets in low-contrast OCT images. Specifically, the heatmap generation network employs depth-separable convolutions to identify key locations, such as the boundaries of the anterior and posterior corneal surfaces and the lens. Subsequently, a confidence prediction head evaluates the reliability of each detected point, thereby mitigating the interference from noise and artifacts commonly found in OCT images, as described in Equation (7).

(7) $$F_{att}=(\mathrm{Sigmoid}(\mathrm{Conv}(\mathrm{DSConv}(X)))\;⊗\;\mathrm{Sigmoid}(\mathrm{Conv}(\mathrm{AdaptiveAvgpool}2d(X))))\;⊗\;X$$

where ⊗ denotes element-wise multiplication, and **AdaptiveAvgpool2d**(·) is 2D adaptive average pooling. This formula integrates spatial attention for landmark localization and channel attention for key feature enhancement.

The coordinate regression head provides high-precision accuracy, while the feature aggregation module generates spatial attention maps based on detected anatomical landmarks, thereby enhancing the representation of features in relevant regions. Based on these enhanced features, this study constructs three parallel detection heads to output pose point results, as shown in Equation (8).

(8) $$\left\{\begin{array}{c} H_{\mathrm{heat}}=\mathrm{DSConv}\;(F_{\mathrm{att}})\\ C_{\mathrm{cont}}=\mathrm{Conv}\;(F_{\mathrm{att}})\\ P_{\mathrm{coord}}=\mathrm{Conv}\;(F_{\mathrm{att}}) \end{array}\right.$$

where $H_{\mathrm{heat}}$ is the keypoint heatmap, $C_{\mathrm{cont}}$ is the detection confidence score, and $P_{\mathrm{coord}}$ is the high-precision coordinate regression result for subsequent geometric parameter calculation.

Residual connections are also incorporated to preserve the original information flow and prevent gradient vanishing during deep network training, thereby improving the stability and performance of the model, as formulated in Equation (9).

(9) $$F_{out}=X+\sigma \;(\mathrm{BN}\;(\mathrm{Conv}\;(\mathrm{Concat}\;(F_{\mathrm{multi}},\;F_{\mathrm{att}}))))$$

#### 2.3.2. SABlock Model

**Figure 6:**
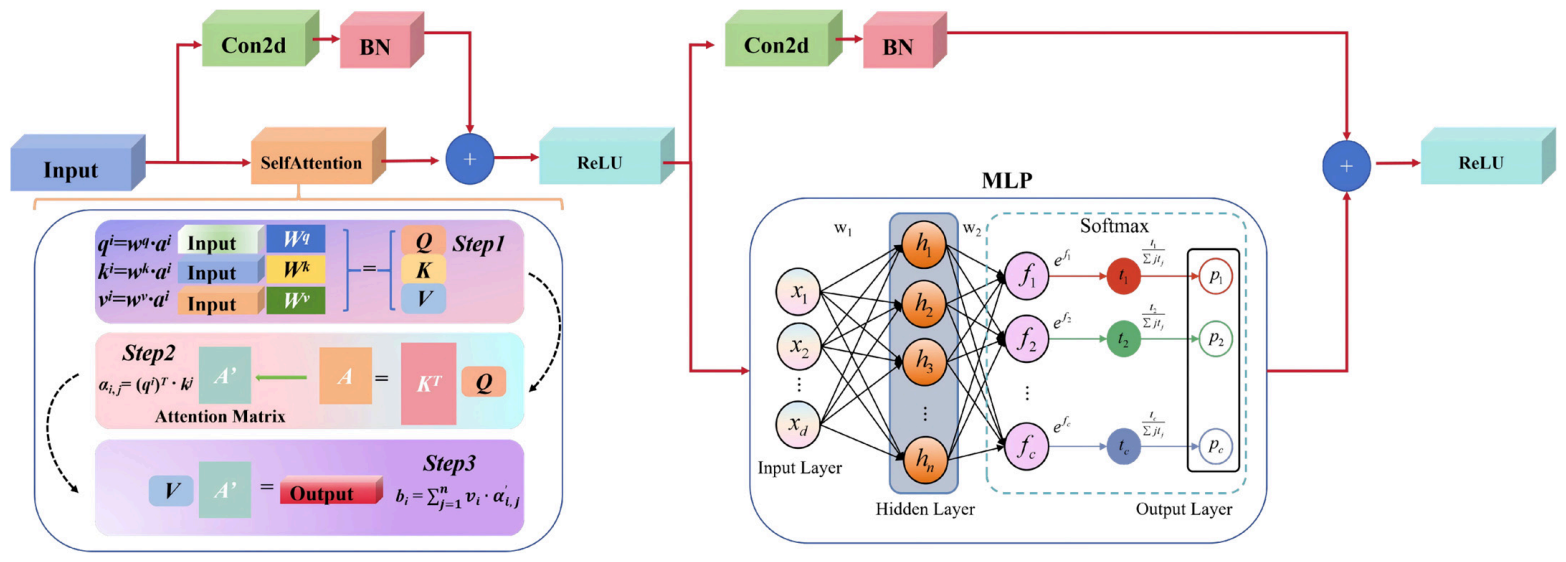
SABlock model network structure diagram. Red solid arrows indicate the forward propagation path of features, orange solid arrows represent residual shortcut connections, and black dashed arrows denote the computational flow of the self-attention mechanism. The left enlarged sub-panel illustrates the three-step self-attention computation process divided by gradient-colored regions, and the blue dashed box in the right MLP module marks the Softmax output layer. The blue circle with a plus sign indicates element-wise residual addition.

Poor pose detection performance in OCT image detection is often caused by a dark background, prompting the design of the SABlock module. The overall network structure is depicted in Figure 6.

Employing a dual-additive residual connection architecture, the SABlock module integrates self-attention with MLP nonlinear transformations to enhance and optimize features for pose point detection in OCT images with complex backgrounds. This design introduces residual connections at the attention and MLP output nodes, effectively preserving the original features while enhancing sensitivity to them. This addresses the critical issue of feature loss in complex environments. The module uses a progressive feature enhancement strategy: first, it suppresses background interference and highlights potential feature regions via self-attention; then, it performs high-level semantic abstraction through the MLP. Dual residual connections ensure continuous propagation of features from low-level textures to high-level semantics, achieving accurate detection of OCT images in complex backgrounds. The computation is defined in Equations (10) and (11):

(10) $$F_{\mathrm{enh}1}\;=\mathrm{ReLU}\;(\mathrm{BN}\;(\mathrm{Conv}2d\;(X))+\mathrm{SelfAtt}\;(X))$$

where **Conv2d(·)** is 2D convolution for local feature extraction, **SelfAtt(·)** is the self-attention mechanism for global dependency modeling, and **ReLU(·)** is the rectified linear unit activation function.

(11) $$F_{out}=\mathrm{ReLU}\;(\mathrm{BN}\;(\mathrm{Conv}2d\;(F_{\mathrm{enh}1}))+\mathrm{MLP}\;(F_{\mathrm{enh}1}))$$

where **MLP(·)** is the multi-layer perceptron for nonlinear semantic transformation, and ***F_out_*** is the final enhanced feature map output by the SABlock module.

#### 2.3.3. C3k2_EVM Model

**Figure 7:**
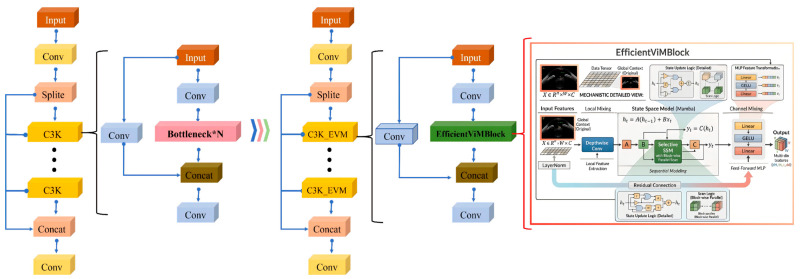
C3k2_EVM model network structure diagram. Blue arrows indicate the forward propagation path of feature maps. The multicolored rightward arrows denote the replacement from the original C3K module to the proposed C3K_EVM module. Functional components are distinguished by different colors, and the red solid box on the right presents a magnified view of the internal architecture of the EfficientViMBlock.

The C3k2 module is the primary feature processor in YOLOv11. Built on a cross-stage local (CSP) architecture, this module achieves multi-scale feature encoding by stacking standard convolutional bottlenecks. However, traditional convolutional bottlenecks have limitations in modeling long-range global dependencies and cannot capture the spatial correlations between dispersed ocular anatomical landmarks in OCT images, resulting in degraded feature representations of pose points in complex, low-contrast backgrounds. To enhance its performance, the EfficientViMBlock has been integrated into the C3k2 module. The EfficientViMBlock forms the core of EfficientViM, a lightweight architecture based on Mamba. As shown in Figure 7, the improved C3k2_EVM module replaces the standard convolutional bottleneck stack in the original C3K2 with the EfficientViMBlock, as shown in Equation (12).

The EfficientViMBlock is a component of this novel visual architecture, which is designed to efficiently capture global dependencies while reducing computational costs. This makes it particularly suitable for environments with limited resources. It uses hidden state mixers and state space duality to optimize computational performance and achieve a better balance between speed and accuracy. The architecture is designed to adapt to varying computational demands while maintaining high efficiency.

(12) $$\begin{array}{c} F_{\mathrm{int}}=\mathrm{Conv}\;(X),\\ \left[F_{\mathrm{main-evm},\;}F_{\mathrm{short-evm}\;}\right]=\mathrm{Split}\;(F_{\mathrm{int}}),\\ F_{\mathrm{main-evm}\;}=\mathrm{EfficientViMBlock}\;(F_{\mathrm{main-evm}\;}),\;\;\;\;\;\;\;\;\;\;\;\;\;\;\;\;\;\;\;\;\;\;\;\;\;\;\;\;\;\;\;\;\;\\ F_{\mathrm{short-evm}\;}=\mathrm{Conv}\;(F_{\mathrm{short-evm}\;}),\\ F_{\mathrm{out}}=\mathrm{Conv}\;(\mathrm{Concat}\;(F_{\mathrm{main-evm},\;}F_{\mathrm{short-evm}\;})) \end{array}$$

## 3. Results

### 3.1. Ablation Study

The present study adopted a methodology of progressively introducing individual modules in order to evaluate the performance of the improved pose point detection model in detecting key anterior chamber geometric parameters (such as IA and anterior chamber depth) in OCT images. Utilizing YOLOv11n_Pose as the baseline model, a comparative and analytical approach was adopted to assess the performance enhancement resulting from the incorporation of each module and diverse module combinations. This method enabled the identification of the individual contributions of each module, as well as the synergistic effects. All models were trained and tested using the same dataset, evaluation metrics, and experimental settings; see Table 2 for details. The presence of a circle (◯) indicates that the module was not added, while the presence of a triangle (▲) indicates that the module was added.

The baseline YOLOv11n pose model yields a mean average precision of 85.5% at mAP@0.5 and 78.5% at mAP@0.5-0.95, with 2.70 million parameters. Replacing the standard convolutions with NewConv improves the precision at 0.5 to 88.2% and marginally reduces parameters to 2.65 million, indicating more effective multi-scale feature representation without computational overhead. The independent integration of the SABlock notably increases the strict interval precision to 81.3%. However, this attention mechanism introduces a substantial computational cost, raising the parameter count to 3.24M. This observation highlights an inherent architectural trade-off between fine-grained localization accuracy and model complexity. To address this parameter expansion, the C3k2 EVM module was incorporated. Independent deployment of C3k2 EVM compresses the baseline model to 2.39M parameters while improving accuracy, demonstrating its efficacy in spatial redundancy reduction.

Finally, the simultaneous integration of all three modules into the NSE YOLO architecture achieves a mean average precision of 90.7% at a 0.5 threshold and 85.1% at the 0.5 to 0.95 interval, constrained within 2.86M parameters. Compared to intermediate configurations, the complete model effectively mitigates the parameter burden introduced by the attention mechanism while maximizing localization precision. These results confirm the structural complementarity of the proposed decoupled design in balancing high-precision accuracy and computational cost.

**Figure 8:**
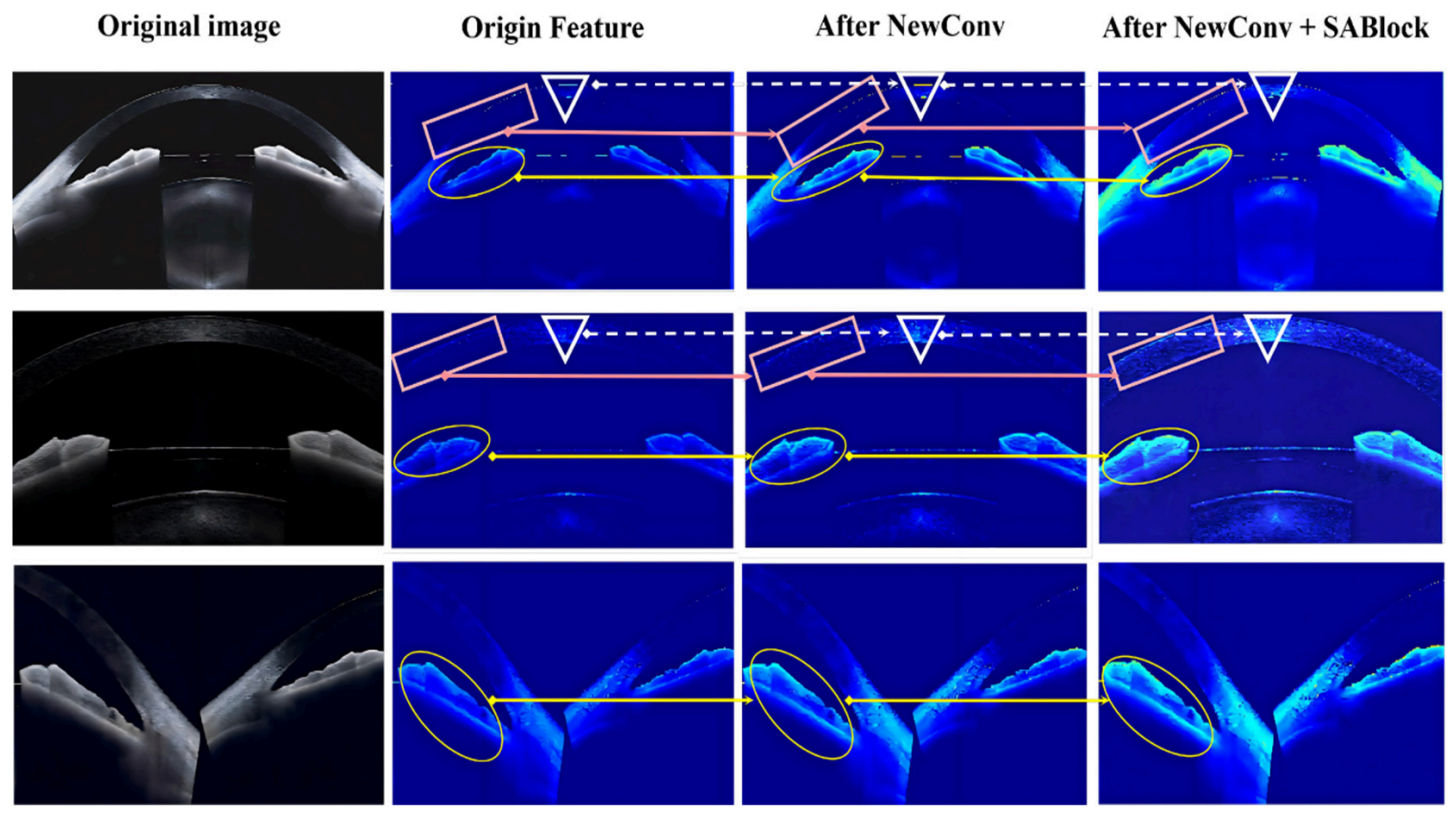
Influence of NewConv and SABlock on feature extraction: The triangular region is indicative of the central area of the cornea and serves as a key reference point for measuring anterior chamber depth. The rectangular region represents the peripheral area of the cornea and forms part of the IA. Colored horizontal arrows indicate the correspondence of identical labeled anatomical regions across different feature extraction stages, and denote the direction of progressive feature enhancement. The elliptical region represents the scleral area, which, in conjunction with the cornea, forms the IA. The integration of the NewConv module has led to significant advancements in the localization of feature details, while the SABlock module has been developed to address the issue of low contrast by suppressing background noise.

Figure 8 illustrates the feature attention heatmaps to evaluate the internal decision logic of the proposed framework. The baseline YOLOv11n architecture presented in the middle column exhibits significant vulnerability to the intrinsic speckle noise and low-contrast backgrounds typical of optical coherence tomography images. The high activation regions indicated by warm color tones are widely dispersed and frequently misaligned with the actual anatomical landmarks. This observation confirms that standard convolution operations struggle to effectively distinguish target features from background artifacts, leading to localization instability and potential coordinate drift. In contrast, the NSE YOLO framework in the right column demonstrates a highly concentrated and precise attention distribution. The activation peaks are tightly anchored to the targeted iridocorneal angles and anterior chamber boundaries, providing objective visual evidence of its superior spatial focus and high-precision localization reliability.

**Figure 9:**
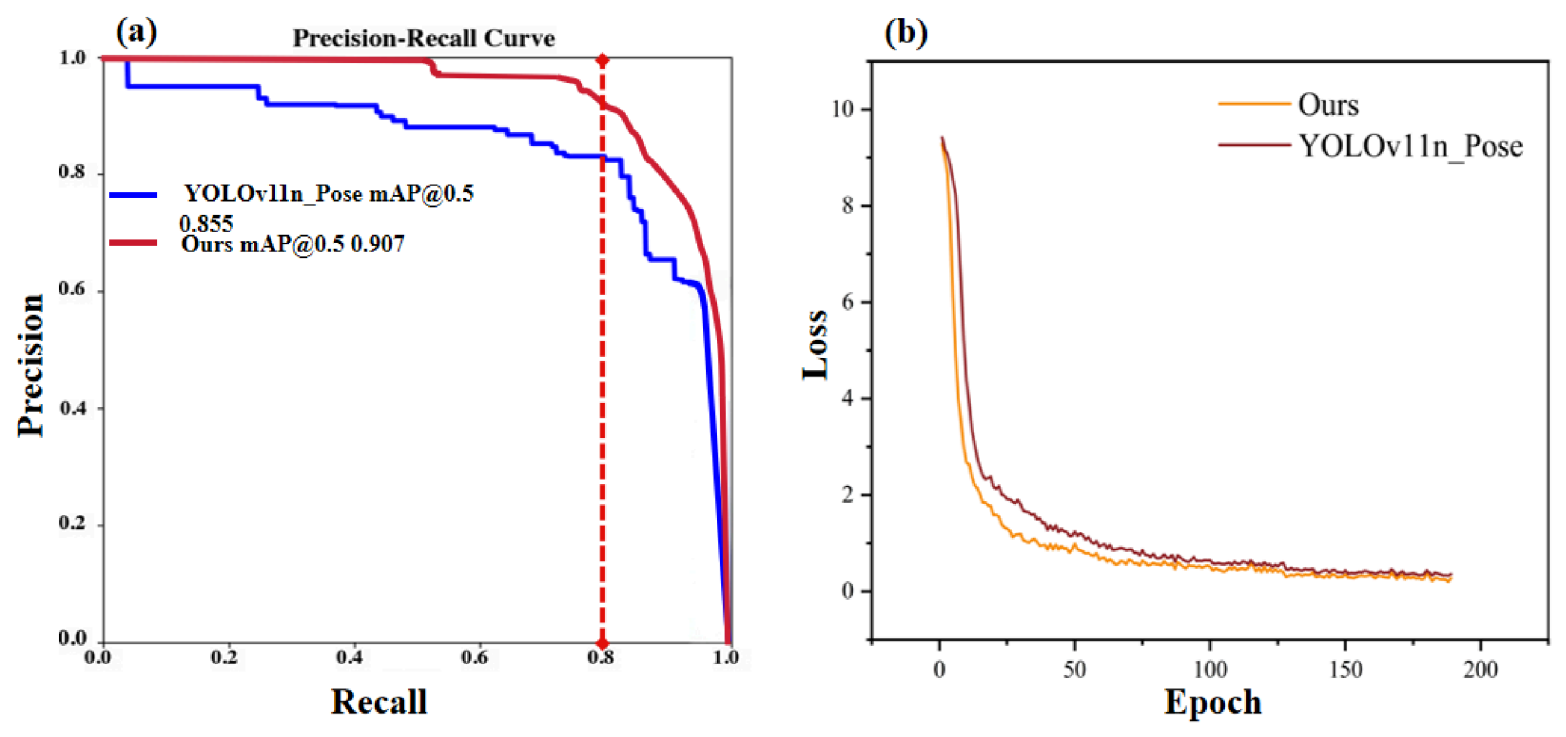
Comparative performance analysis of the NSE YOLO (Ours) and YOLOv11n_Pose. (**a**) Precision–recall curves with corresponding mAP@0.5 values, where the red vertical dashed line serves as an alignment reference for visually comparing the precision gap between the two models at the same recall level. (**b**) Training loss curves illustrating the convergence behavior across epochs.

As illustrated by the precision–recall curve in Figure 9a, the proposed model achieves an mAP@0.5 of 0.907, higher than the 0.855 of the baseline YOLOv11n_Pose network. As the recall increases, the precision of the proposed method decays more slowly, indicating that the improved network structure better balances the false negative and false positive rates in the anatomical landmark detection of anterior segment images. Meanwhile, the training loss curves in Figure 9b show that the loss value of the proposed model decreases faster in the early stages of iteration, remains lower than that of the baseline network throughout the training cycle, and ultimately converges to a lower level. This gradient update process reflects that the proposed modifications improve the feature learning efficiency of the network.

### 3.2. Comparisons with Previous Methods

All comparator models were trained and evaluated under strictly consistent conditions, including the same patient-partitioned dataset, identical data augmentation pipeline, and unified training hyperparameters. YOLO-series baselines adopt the same keypoint detection head architecture as the proposed model for full-task comparison. Two-stage detectors are limited to bounding-box level comparison due to inherent architectural constraints. This configuration ensures the transparency and fairness of cross-model performance comparison.

To evaluate the proposed NSE YOLO framework, we conducted quantitative comparisons against state-of-the-art models, including two-stage architectures such as FastRCNN, Cascade-RCNN and single-stage detectors, including YOLOv5n, YOLOv8n, YOLOv8s, on our dataset. The comparative results in Table 3 indicate that traditional two-stage models suffer from heavy parameter burdens, making them suboptimal for clinical deployment. While baseline YOLO models improve efficiency, they frequently exhibit localization drift at low-contrast tissue interfaces. In contrast, our NSE YOLO achieves an optimal balance. It yields a superior mean average precision of 90.7% at a 0.5 threshold and 85.1% within the strict 0.5 to 0.95 interval. Compared to the baseline YOLOv11n pose, it realizes an absolute precision gain of 5.6% while maintaining a highly compact envelope of merely 2.86 million parameters. This performance leap is structurally driven by the synergistic noise suppression and feature amplification of the NewConv and SABlock modules, establishing a robust and lightweight framework for high-fidelity automated clinical analysis.

### 3.3. Experimental Visualization

**Figure 10:**
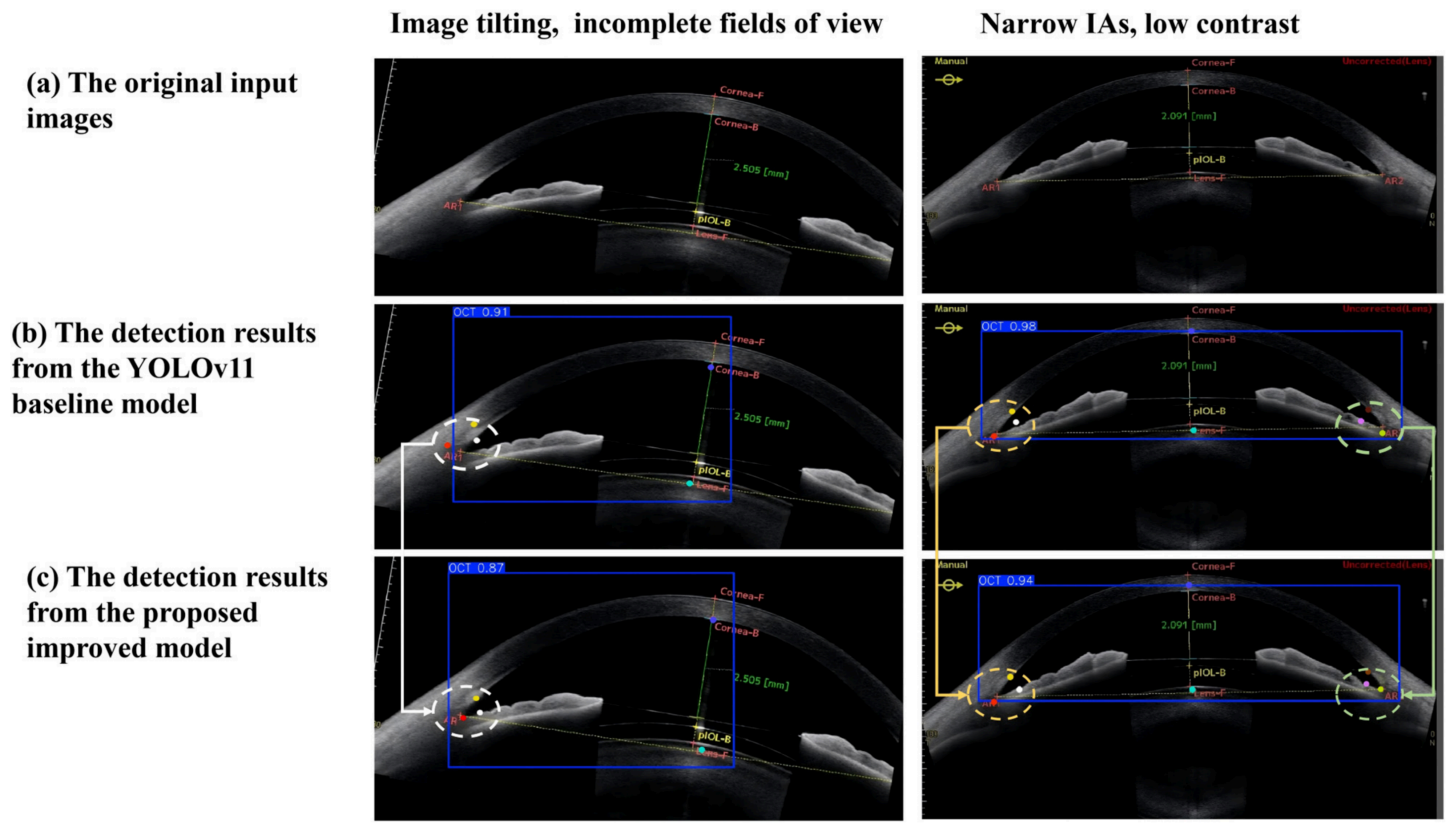
Comparison of detection results: (**a**) the original input images; (**b**) the detection results from the YOLOv11 baseline model; (**c**) the detection results from the proposed improved model. The elliptical dashed line is a representation of the IAs region, as delineated by the pose points. The annotated landmarks define the anterior chamber region (blue box), left IA (red, white, and light yellow points), right IA (purple, yellowish, green, and brown points), and anterior chamber depth (distance between the blue and light green points).

To evaluate the practical performance under complex clinical scenarios such as low contrast, narrow IAs, image tilting, and incomplete fields of view, Figure 10 visually compares the detection outcomes of the baseline YOLOv11 and the proposed model. While the baseline architecture frequently suffers from localization drift and ambiguous target delineation under these degraded imaging conditions, the proposed model accurately localizes key anatomical landmarks, including the bilateral IAs and anterior chamber depth, maintaining clear structural boundaries. This qualitative stability is directly attributed to the designated architectural components: NewConv optimizes multi-scale feature fusion, SABlock enhances spatially discriminative features against background noise, and C3k2_EVM provides efficient global contextual modeling. Ultimately, these visual findings corroborate the quantitative metrics, confirming the framework’s reliability and translational potential for automated clinical image analysis.

### 3.4. Cross-Validation Robustness

Because the held-out test set constitutes only 10% of the data, single-split performance estimates may be sensitive to the particular partition. To assess robustness, we additionally performed patient-level five-fold cross-validation, with data augmentation applied independently within the training subset of each fold. Table 4 summarizes the pooled results for the baseline (YOLOv11n-Pose) and the proposed NSE YOLO.

Across all five folds, NSE YOLO consistently outperformed the baseline (mAP@0.5 +3.0%, mAP@0.5:0.95 +5.0%), indicating that the improvement is not contingent on any single partition. Moreover, the standard deviation of NSE YOLO was markedly smaller (mAP@0.5:0.95 decreasing from 0.0480 to 0.0238, a reduction of more than half), demonstrating that the proposed modules enhance not only accuracy but also cross-partition stability. The cross-validation means are directionally consistent with the single-split results reported above; the modestly lower values reflect averaging over five distinct partitions and confirm that the main results do not depend on a single fortunate split.

### 3.5. Clinically Oriented Quantitative Evaluation

Although mAP@0.5 and mAP@0.5:0.95 are informative computer-vision metrics, they do not directly reflect clinically meaningful measurement error. We therefore supplemented the evaluation with clinically interpretable metrics computed on the test set.

Table 5 summarizes per-landmark localization error on the test set, expressed in pixel units and NME. The eight anatomical landmarks show an overall mean error of 10.53 pixels, corresponding to an overall NME of 1.02%. Error magnitude varies across anatomical regions. IA landmarks exhibit larger deviation, with the left angle apex showing the highest error (NME = 1.52%). Landmarks for ACD have the smallest localization deviation, with NME values below 0.75% for both corneal posterior and lens anterior surfaces.

Table 6 presents clinically meaningful measurement errors and the proportion of cases falling within accepted tolerance thresholds. For ACD, the MAE was 0.118 mm, with 80.0% of samples within the 0.2 mm clinical threshold, and a median error of 0.109 mm. For IA measurements, MAE was 5.10° for the left IA and 4.90° for the right IA. The within-threshold rate was 73.3% for the left IA and 60.0% for the right IA. Median absolute errors for both IAs fall below the 5-degree threshold, indicating that more than half of the samples meet clinical acceptability. The slightly higher overall error is driven primarily by a small subset of extreme cases with severely narrow IAs or substantial imaging artifacts.

**Figure 11:**
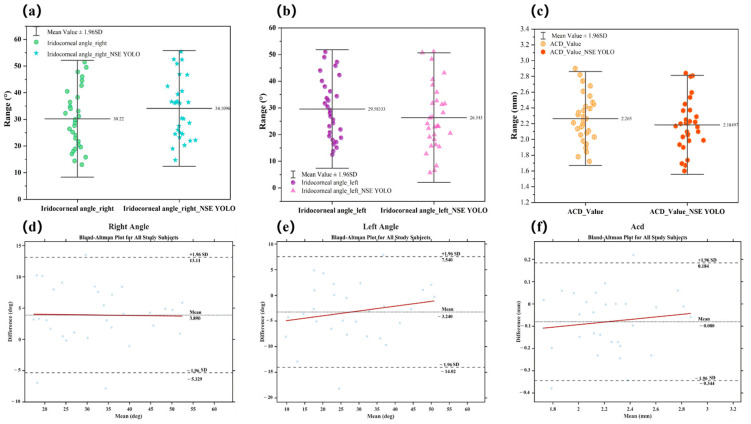
Measurement distributions and Bland–Altman analyses of NSE-YOLO and expert measurements. Panels (**a**–**c**) show the right angle, left angle, and anterior chamber depth, while panels (**d**–**f**) show the corresponding Bland–Altman plots. Differences were calculated as NSE-YOLO minus expert measurements. Dashed lines indicate the 95% limits of agreement, and red lines indicate the linear regression trends.

Bland–Altman analysis showed small systematic differences between NSE-YOLO and expert measurements, with negative biases for the left angle and ACD and a positive bias for the right angle. The distributions and limits of agreement are presented in Figure 11, while the corresponding numerical estimates and 95% confidence intervals are summarized in Table 7. Table 7 summarizes the Bland–Altman analysis between the NSE-YOLO and expert measurements. The mean bias for ACD was −0.08 mm, with a 95% confidence interval of −0.130 to −0.030 mm and 95% limits of agreement of −0.344 to 0.184 mm. The mean bias for the left angle was −3.24°, with a 95% confidence interval of −5.29° to −1.19° and 95% limits of agreement of −14.02° to 7.54°. For the right angle, the mean bias was 3.89°, with a 95% confidence interval of 2.13° to 5.65° and 95% limits of agreement of −5.33° to 13.11°.

Figure 11 presents the measurement distributions and Bland–Altman analyses for the left and right IAs and ACD obtained by the experts and NSE-YOLO. The mean right-angle measurement increased from 30.22° for the expert measurements to 34.11° for NSE-YOLO, whereas the mean left-angle measurement decreased from 29.58° to 26.34°. The mean ACD decreased from 2.265 to 2.185 mm. In the Bland–Altman plots, the differences for the left angle and ACD were predominantly distributed below zero, whereas those for the right angle were predominantly distributed above zero, consistent with the directions of bias reported in Table 7. The distributions of paired differences and the corresponding upper and lower 1.96 SD limits are shown in panels d–f.

A stratified sensitivity analysis was performed to examine whether aggregate error is concentrated in specific morphological subgroups. Test images were grouped by ground-truth magnitude of anterior chamber depth and iridocorneal angle, with mean absolute error recomputed within each stratum. Results are summarized in Table 8.

ACD error remains stable across shallow, intermediate and deep strata, with no abrupt fluctuation. IA measurement error increases gradually from open to narrow configurations, following a near-monotonic trend. Larger error in narrow-IA and shallow-chamber subgroups aligns with reduced visibility of the angle recess under these conditions, consistent with patterns observed in the failure case analysis. The smooth gradient of error across strata indicates that model performance degrades gracefully rather than collapsing abruptly in any single morphological subgroup.

To contextualize model performance against the inherent reliability of the reference standard, inter-observer variability among the three annotators was quantified on the same test set. For each landmark, mean pairwise Euclidean distance between independent annotations was computed and normalized using the same reference length as model NME. Results are shown in Table 9.

Overall inter-observer NME was 0.95%, which is comparable in magnitude to the model localization error of 1.02%. This pattern was consistent across both angle-related and ACD-related landmark groups: model NME was 0.08 percentage points higher than inter-observer variability for IA landmarks (1.12% vs. 1.04%), and 0.06 percentage points higher for ACD landmarks (0.75% vs. 0.69%). These results indicate that the model’s localization performance has approached the level of inter-observer agreement among human annotators. Part of the observed model error, particularly for angle landmarks in low-contrast or anatomically ambiguous images, may reflect uncertainty in the reference annotations rather than being solely attributable to model limitations.

### 3.6. Failure Case Analysis

**Figure 12:**
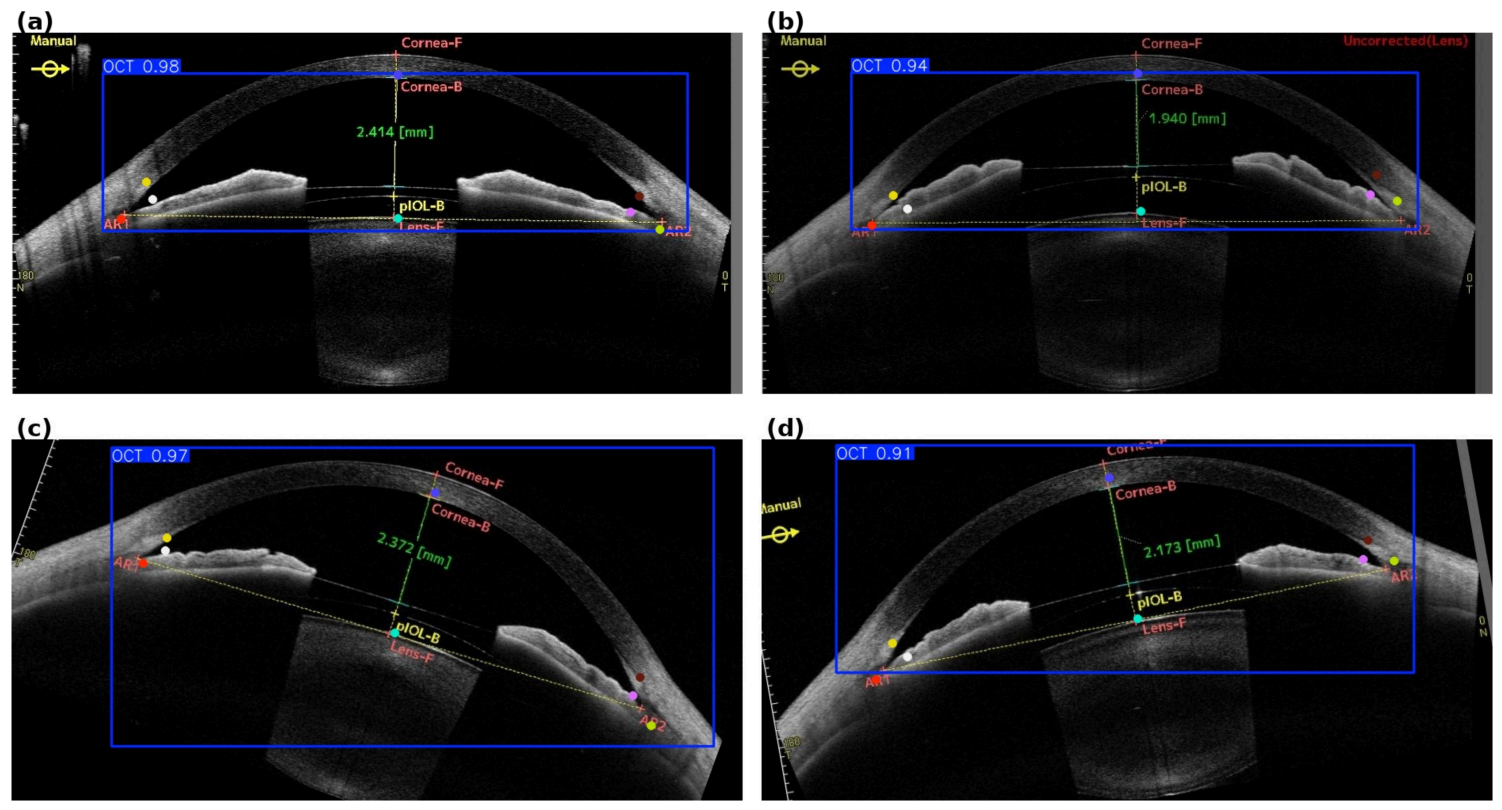
Representative failure cases of the proposed NSE YOLO framework under extreme imaging conditions. (**a**) Poor structural visibility with vertical banding artifacts; (**b**) an extremely narrow iridocorneal angle in which the angle recess is nearly collapsed into a single line; (**c**) an extremely oblique section combined with low contrast; (**d**) mixed extreme conditions combining a narrow angle, low contrast, and tilt. Under these conditions, the localization of angle-related landmarks becomes unstable, reflecting the intrinsic difficulty of both automated and manual annotation in such cases.

To characterize the boundaries of the proposed method, we examined representative cases in which localization was unreliable (Figure 12). These correspond precisely to the most challenging clinical conditions: images with poor structural visibility and vertical banding artifacts (Figure 12a), extremely narrow angles in which the angle recess collapses into a near-linear structure (Figure 12b), extremely oblique sections combined with low contrast (Figure 12c), and mixed extreme conditions that simultaneously present a narrow angle, low contrast, and tilt (Figure 12d). In these scenarios, the attenuation or ambiguity of the scleral spur and angle recess lowers the confidence of the keypoint heads and can induce coordinate drift, particularly for the angle-related landmarks. Such cases are clinically important because they represent precisely the conditions under which automated landmark localization—and indeed manual annotation—is inherently most difficult, and they delineate the scenarios in which automated measurements should be interpreted with caution or subjected to manual review.

## 4. Discussion

This paper proposes a novel algorithm specifically designed for the accurate and efficient localization of key regions in anterior segment OCT images, building upon the YOLOv11 framework. This approach addresses the challenges posed by the inherent limitations of current OCT imaging, including unavoidable speckle noise, low contrast, projection artifacts, field-of-view and operational constraints, and a heavy reliance on subjective human judgment [23,27,35].

In order to address the challenges identified, this study proposes the incorporation of a dual-branch pose detection module (NewConv) and a self-attention module (SABlock) with a dual-additive residual architecture into the backbone structure. Furthermore, a module based on EfficientViM (C3k2_EVM) has been integrated into the neck structure. These enhancements have been shown to significantly improve the model’s ability to identify small objects in complex scenes, achieving an mAP@0.5 of 90.7% and a mAP@0.5-0.95 of 85.1%, representing improvements of 5.2% and 6.6%, respectively, over the baseline model.

This enhanced feature representation is structurally attributed to the synergistic integration of the NewConv and SABlock modules. The NewConv module utilizes a dual-branch architecture to drive early feature convergence. Its multi-scale extraction path aggregates local textures via 1 × 1 convolutions, medium-scale shape variations via 3 × 3 convolutions, and global deformations through 5 × 5 depthwise separable convolutions. Simultaneously, the SABlock module addresses the dark-background interference through a progressive enhancement strategy. By combining self-attention mechanisms with nonlinear multilayer perceptron transformations, the module actively suppresses background noise while highlighting potential landmark regions. Crucially, the dual-additive residual connections deployed across these nodes prevent the degradation of original feature information, ensuring a continuous representation flow from low-level textures to high-level semantics. This decoupled design effectively maximizes landmark localization accuracy while strictly regulating the influence of complex clinical noise. Concurrently, the Efficient ViM module drastically prunes redundant feature channels, compressing the parameter count to an ultra-compact level of under three million without sacrificing spatial resolution. In recent years, a variety of models have been proposed for object detection and localization, particularly for application on OCT images [40,41,42,43,44,45,46,47]. However, from a clinical translation perspective, most of these algorithms require substantial computational resources and lengthy processing times, which limits their feasibility in clinical settings. The proposed algorithm in this study demonstrates a superior equilibrium between localization accuracy and computational efficiency, as evidenced in Table 2 and Table 3. This framework demonstrates a high degree of compatibility with ophthalmic edge devices that are constrained in terms of resources, thereby effectively supporting real-time automated clinical assessment.

Choi et al. [60] reviewed the application of artificial intelligence in refractive surgery and showed that previous approaches in phakic IOL implantation have mainly focused on patient selection, ICL sizing, postoperative vault and anterior chamber angle prediction, and refractive outcome estimation, frequently using structured ocular biometric data. In this context, NSE YOLO addresses a distinct but complementary stage of the clinical workflow. Rather than predicting surgical parameters or postoperative outcomes from pre-extracted variables, it directly localizes anatomical landmarks in AS-OCT images and derives the bilateral iridocorneal angles and anterior chamber depth. The framework is specifically designed to maintain localization performance in the presence of speckle noise, low contrast, and indistinct tissue interfaces while retaining a compact architecture of 2.86 million parameters. This image-to-measurement approach extends the current AI workflow by automating the extraction of quantitative anterior segment parameters that can support postoperative assessment and provide standardized anatomical inputs for subsequent planning or prediction models.

The Bland–Altman analysis showed that the differences between NSE-YOLO and expert measurements were parameter-dependent. The mean bias for ACD was −0.08 mm, indicating slight underestimation by the model. This bias remained below the predefined error criterion of 0.2 mm, and the limits of agreement were narrower than those observed for the angle measurements, indicating greater stability in ACD localization. The mean biases for the left and right angles were −3.24° and 3.89°, respectively, both within the 5° error criterion. The opposite directions of bias suggest that the angular measurements were not affected by a uniform systematic shift, but may instead be influenced by side-specific anatomical morphology and landmark visibility. Although the confidence intervals of the biases did not include zero, indicating detectable fixed differences between NSE-YOLO and expert measurements, their statistical significance should be interpreted together with the magnitude of the bias. Overall, NSE-YOLO maintained good mean-level agreement for both ACD and iridocorneal angle measurements. Agreement was more stable for ACD, whereas angle measurements showed greater individual variability and side-dependent deviations. These findings support the use of NSE-YOLO for automated quantitative assessment of anterior chamber structures after ICL implantation and suggest that side-specific calibration may further improve angular measurement consistency.

At the landmark level, the error distribution follows the expected gradient of anatomical difficulty: angle landmarks with higher structural variability show larger deviation, while the well-defined boundaries for ACD measurement yield higher accuracy. The overall model performance approaches inter-observer agreement, indicating that a non-negligible portion of the measured error stems from inherent uncertainty in the reference standard rather than model deficiency alone.

It is also worth noting that expert annotation is not inherently free from error. Under conditions of low image contrast, blurred structural boundaries, or elevated background noise, human annotators are susceptible to systematic misjudgment, as perceptual ambiguity at the tissue interface can lead to inconsistent landmark placement across observers and even within the same observer across sessions. This vulnerability is precisely the problem NSE YOLO was designed to address. By learning feature representations directly from imaging data rather than relying on subjective visual interpretation, the model demonstrates a capacity to maintain measurement consistency in scenarios where human performance may paradoxically deteriorate. In this context, the mean differences observed in the Bland–Altman analyses should not be interpreted solely as algorithmic error; a non-negligible portion of the measured discrepancy may, in fact, originate from instability in the reference annotations themselves, particularly in cases involving ambiguous anterior segment morphology. This possibility introduces an inherent ceiling on the agreement metrics that can be achieved against a human-derived ground truth and underscores the need for future validation frameworks that incorporate inter-annotator reliability assessments alongside automated performance evaluation.

Several limitations should be acknowledged. First, all data originated from a single center and a single device (CASIA2 AS-OCT); consequently, the present study does not include an independent multi-device or multi-center external cohort, and the five-fold cross-validation reported above characterizes internal robustness rather than cross-distribution generalization. Although the dataset already spans low-contrast, narrow-angle, boundary-ambiguous, non-standard-section, and post-ICL cases—and thus captures a degree of data heterogeneity—external validation on device-independent, time-independent, and multi-center cohorts remains necessary and is planned for future work. Second, the extended Bland–Altman analysis revealed a mild systematic bias in the angle measurements (slight underestimation of the left angle and slight overestimation of the right angle); reducing this directional angle bias is an explicit target for further improvement. The mean absolute error of angle measurement is close to the 5° clinical threshold, with performance approaching the inter-observer variability of human annotation. Part of the observed measurement discrepancy derives from inherent ambiguity in landmark placement under low-contrast and narrow-angle conditions. At its current performance level, the model can serve as an auxiliary tool for batch preprocessing and preliminary screening of AS-OCT images. Manual review remains recommended for borderline or clinically high-risk cases to ensure diagnostic accuracy. Third, a full quantitative inter- and intra-observer variability study was not performed, and future validation frameworks should incorporate inter-annotator reliability assessment alongside automated performance evaluation. Fourth, the current model was validated only on AS-OCT images obtained after ICL implantation. Its generalizability to preoperative phakic eyes, pseudophakic eyes after cataract surgery, and other anterior segment conditions remains unconfirmed. Targeted external validation for each clinical subgroup will be conducted in subsequent work.

## 5. Conclusions

To address the challenges of labor-intensive annotation, speckle noise, low-contrast interfaces, and complex backgrounds in AS-OCT images, this study developed NSE YOLO, an enhanced YOLOv11 framework integrating multi-scale feature extraction, attention-based background suppression, and efficient global contextual modeling. The proposed architecture improved landmark localization performance while retaining a compact model structure, with principal findings as follows:

(1) **Multi-scale feature representation with NewConv**: The dual-branch parallel NewConv module integrates features with different receptive fields. Ablation results showed it improved baseline localization performance without increasing parameter count, supporting effective representation of small, boundary-ambiguous anatomical landmarks in noisy AS-OCT images.
(2) **Background suppression with SABlock**: The SABlock combines self-attention, nonlinear feature transformation, and dual residual connections. Its incorporation improved localization stability under low-contrast and background-interference conditions, indicating that contextual feature enhancement contributes to more robust landmark representation.
(3) **Computational efficiency with C3k2_EVM**: The Mamba-based EfficientViMBlock embedded in the C3k2 module captures global contextual information while reducing redundant feature processing. The full NSE YOLO model achieved improved localization accuracy with 2.86 million parameters, demonstrating a favorable balance between performance and model complexity in the present experimental setting.
(4) **Clinical measurement agreement**: NSE YOLO achieved an mAP@0.5 of 90.7% and an mAP@0.5:0.95 of 85.1%, outperforming the YOLOv11n-Pose baseline. Patient-level five-fold cross-validation further confirmed the consistency of performance improvement across internal data partitions. Landmark-level error, clinical parameter error, and Bland–Altman analyses demonstrated favorable and reliable agreement between model-derived measurements and the expert reference standard for anterior chamber depth. For bilateral iridocorneal angles, the model achieves localization performance comparable to inter-observer variability among human annotators, providing clinically usable auxiliary measurements with remaining room for accuracy improvement.

## Figures and Tables

**Table 1:** Experimental parameters.

Parameter	Setup
Epochs	200
Batch size	32
Optimizer	SGD
Learning rate	0.01
Momentum	0.937
Weight decay	0.0005

**Table 2:** Performance comparison of different detection algorithms.

Model	+NewConv	+SABlock	+C3k2_EVM	mAP @0.5(%)	mAP @0.5-0.95(%)	Parmas/10^6^
YOLOv11n_Pose	◯	◯	◯	85.5	78.5	2.70
1	▲	◯	◯	88.2	80.6	2.65
2	◯	▲	◯	87.7	81.3	3.24
3	◯	◯	▲	87.3	80.2	2.39
4	▲	▲	◯	90.0	82.9	2.94
5	◯	▲	▲	88.6	82.4	2.87
6	▲	◯	▲	89.2	81.8	2.77
NSE YOLO (Ours)	▲	▲	▲	90.7	85.1	2.86

**Table 3:** Comparison with previous methods.

Model	mAP @0.5(%)	mAP @0.5-0.95(%)	Parmas/10^6^
YOLOv11n_Pose	85.5	78.5	2.70
YOLOv5n	82.9	55.3	2.78
YOLOv8n	83.4	56.4	2.83
YOLOv8s	86.1	60.7	7.73
FastRCNN	80.1	74.5	41.10
Cascade-RCNN	79.2	73.1	69.44
NSE YOLO (Ours)	90.7	85.1	2.86

**Table 4:** Five-fold cross-validation results (mean ± standard deviation).

Model	mAP@0.5	mAP@0.5:0.95	Precision	Recall
YOLOv11n-Pose (baseline)	0.8541 ± 0.0519	0.8021 ± 0.0480	0.8593 ± 0.0309	0.8608 ± 0.0378
NSE YOLO (ours)	0.8842 ± 0.0227	0.8522 ± 0.0238	0.8628 ± 0.0312	0.8765 ± 0.0244

**Table 5:** Per-landmark localization error on the test set.

Landmark	Mean px	Std px	Median px	NME (%)
Left IA apex	15.68	7.01	16.52	1.52
Left IA upper	11.57	6.88	12.36	1.12
Left IA lower	12.02	6.69	9.71	1.17
Right IA apex	10.01	6.39	10.17	0.97
Right IA upper	11.19	7.20	8.71	1.09
Right IA lower	8.72	8.02	7.77	0.84
ACD top (Cornea-B)	7.49	4.80	7.72	0.73
ACD bottom (Lens-F)	7.60	4.93	7.52	0.74
Overall (8 pts)	10.54	—	—	1.02

**Table 6:** Clinical parameter error and proportion of cases within accepted thresholds.

Parameter	MAE	Std	Median	Within Threshold	Threshold
ACD (mm)	0.118	0.072	0.109	80.0%	≤0.2 mm
Left IA (°)	5.10	3.80	3.72	73.3%	≤5.0°
Right IA (°)	4.90	3.62	4.61	60.0%	≤5.0°

**Table 7:** Extended Bland–Altman analysis with 95% confidence intervals.

Parameter	Bias	95% CI of Bias	LoA
ACD (mm)	−0.08	[−0.130, −0.030]	[−0.344, +0.184]
Left angle (°)	−3.24	[−5.29, −1.19]	[−14.02, +7.54]
Right angle (°)	+3.89	[+2.13, +5.65]	[−5.33, +13.11]

**Table 8:** Stratified sensitivity analysis: MAE within subgroups defined by the ground-truth magnitude of the ACD and of the iridocorneal angle.

Stratum	Proportion in Test Set	MAE
ACD—shallow (<2.0 mm)	20%	0.135 mm
ACD—intermediate (2.0–2.5 mm)	60%	0.11 mm
ACD—deep (>2.5 mm)	20%	0.12 mm
IA—narrow (<20°)	25%	5.90°
IA—intermediate (20–35°)	45%	4.95°
IA—open (>35°)	30%	4.20°

**Table 9:** Comparison of the model’s per-region localization error with the inter-observer variability of the three annotators on the same test images (NME, normalized mean error).

Landmark Group	Model NME (%)	Inter-Observer NME (%)
IA landmarks (6 pts)	1.12	1.04
ACD landmarks (2 pts)	0.74	0.69
Overall (8 pts)	1.02	0.95

## Data Availability

The datasets used and/or analyzed in this current study are available from the corresponding author on reasonable request.
